# Various *Wolbachia* genotypes differently influence host *Drosophila* dopamine metabolism and survival under heat stress conditions

**DOI:** 10.1186/s12862-017-1104-y

**Published:** 2017-12-28

**Authors:** Nataly Е. Gruntenko, Yury Yu. Ilinsky, Natalya V. Adonyeva, Elena V. Burdina, Roman A. Bykov, Petr N. Menshanov, Inga Yu. Rauschenbach

**Affiliations:** 1 The Institute of Cytology and Genetics of SB RAS, Novosibirsk, 630090 Russia; 2 Novosibirsk State University, Novosibirsk, Russia; 3 School of Life Sciences Immanuel Kant Baltic Federal University, Kaliningrad, Russia; 4 Novosibirsk State Technical University, Novosibirsk, Russia

**Keywords:** *Drosophila melanogaster*, *Wolbachia*, Dopamine metabolism, Heat stress, Stress resistance

## Abstract

**Background:**

One of the most widespread prokaryotic symbionts of invertebrates is the intracellular bacteria of *Wolbachia* genus which can be found in about 50% of insect species. *Wolbachia* causes both parasitic and mutualistic effects on its host that include manipulating the host reproductive systems in order to increase their transmission through the female germline, and increasing the host fitness. One of the mechanisms, promoting adaptation in biological organisms, is a non-specific neuroendocrine stress reaction. In insects, this reaction includes catecholamines, dopamine, serotonin and octopamine, which act as neurotransmitters, neuromodulators and neurohormones. The level of dopamine metabolism correlates with heat stress resistance in *Drosophila* adults.

**Results:**

To examine *Wolbachia* effect on *Drosophila* survival under heat stress and dopamine metabolism we used five strains carrying the nuclear background of interbred Bi90 strain and cytoplasmic backgrounds with different genotype variants of *Wolbachia* (produced by 20 backcrosses of Bi90 males with appropriate source of *Wolbachia*). Non-infected Bi90 strain (treated with tetracycline for 3 generations) was used as a control group. We demonstrated that two of five investigated *Wolbachia* variants promote changes in *Drosophila* heat stress resistance and activity of enzymes that produce and degrade dopamine, alkaline phosphatase and dopamine-dependent arylalkylamine N-acetyltransferase. What is especially interesting, *wMelCS* genotype of *Wolbachia* increases stress resistance and the intensity of dopamine metabolism, whereas wMelPop strain decreases them. *wMel, wMel2* and *wMel4* genotypes of *Wolbachia* do not show any effect on the survival under heat stress or dopamine metabolism. L-DOPA treatment, known to increase the dopamine content in *Drosophila*, levels the difference in survival under heat stress between all studied groups.

**Conclusions:**

The genotype of symbiont determines the effect that the symbiont has on the stress resistance of the host insect.

## Background

The phenomenon of symbiosis can hardly be overestimated: apparently, there is no multicellular organism in nature without symbiotic relations. The crucial issues of symbiosis are the identification of evolutionary stages of establishing interactions between partners and the revealing of molecular mechanisms of these interactions at the cellular and gene level. One of the most widespread prokaryotic symbionts of invertebrates is the intracellular α-proteobacteria *Wolbachia pipientis* that infects no less than a 40% of terrestrial arthropods [1]. *Wolbachia* is maternally transmitted and is able to manipulate host sex determination or reproductive systems in order to help *Wolbachia* spread in host populations [2, 3]. On the other hand, *Wolbachia* infection could be beneficial to its host [4–8]. Differences in the phenotypical manifestations of the infection can be due to singularities of the host organism physiology, including processes of the endocrine regulation of growth, development and fitness. Indeed, there is a lot of research that connects biochemical changes in hosts and phenomenon of host resistance to viral infections with *Wolbachia* symbiont [6, 9–12]. However, molecular mechanisms underlying the basis of *Wolbachia*-host interactions as well as physiological mechanisms by which *Wolbachia* promotes adaptation of the host organism remain largely unknown. There are several model organisms to study these issues on, and *Drosophila melanogaster* is the most studied one.

Field populations of *D. melanogaster* are ubiquitously infected with *Wolbachia* in the frequency range of 30–60% [13–19]. *Wolbachia* symbionts of *D. melanogaster* have monophyletic origin with divergence time ~ 8 Kya [20]. Several lineages/genotypes/strains of *Wolbachia* were identified via different approaches. According to the phylogeny reconstruction of full genome sequences, the symbiont diversity in *D. melanogaster* includes the I-VI and VIII clades [20, 21]. In terms of polymorphism of certain genome markers the six genotypes were revealed, i.e. *wMel, wMel2, wMel3, wMel4, wMelCS, wMelCS2* [18, 22]. Regarding the *Wolbachia* effect or its source (fly stock), several strains were investigated but two of them (wMel and wMelPop) are the most significant, especially in our discourse. Thus, wMel strain is regarded as a common monophyletic group that covers all of the diversity of bacteria isolated from *D. melanogaster*; whereas wMelPop is a certain pathogenic variant of wMel strain that causes early death of flies [23]. These classifications are consistent as follows: *Wolbachia* diversity can be reduced to unique, monophelytic wMel strain [24–26], which can be divided into several genotypes, among which wMelPop strain is just a variant of *wMelCS* genotype. The *wMel* genotype (not to be mixed with wMel strain) includes I-V and VIII clades and is the most widespread [18, 20, 27]. The *wMel2* genotype belongs to VIII clade, *wMel4* – to III clade, *wMelCS* and *wMelCS2* belong to VI clade [18, 20, 21, 27].

One of the physiological mechanisms that promote adaptation and could be potentially influenced by *Wolbachia* is a non-specific neuroendocrine stress reaction. In insects, it includes several components, such as juvenile hormone, ecdysone, insulin and biogenic amines, in particular – dopamine (DA) [28–30]. DA plays three different roles in *Drosophila*: a neurotransmitter passing the nerve impulse through the synaptic cleft; a neuromodulator affecting the neighbouring neurons and modifying neurotransmitter action; and a neurohormone that is transported by the haemolymph and acts remotely [31]. Under the stress the DA level in *Drosophila* rises quickly and steeply, impacting survival [32–34]. The activity of alkaline phosphatase (ALP), an enzyme regulating the pool of DA precursor tyrosine, is shown to decrease under stress following the rise of the DA level that down-regulates it [35, 36]. As to the basal DA level under normal conditions, it is determined, at least particularly, via DA-dependent arylalkylamine N-acetyltransferase (DAT) activity [37, 38]. Here we study an effect of several *Wolbachia* genotypes on *Drosophila* heat stress resistance and the DA metabolism in order to evaluate the role of *Wolbachia* diversity in the symbiont influence on the host adaptability.

## Methods

### *Drosophila melanogaster* strains and rearing

To examine *Wolbachia* effect on physiological and biochemical traits of *D. melanogaster*, the nuclear background of Bi90 isofemale strain and different cytoplasmic backgrounds were used. Bi90 strain was established from wild-caught female of “Bishkek 2004” population and interbred for more than 300 generations, thereby it could be considered a nearly isogenic line. This strain was earlier characterized by *Wolbachia* infection and mtDNA [18, 39, 40]. One pair of flies from Bi90 strain was isolated to get Bi90 branch, which was treated with tetracycline for 3 generations to make *Wolbachia*-free Bi90^T^ strain [6, 27]. Bi90^T^ strain was used in making conplastic strains and as a control in experiments.

Five *D. melanogaster* strains with different *Wolbachia* infections were used in the study: Bi90 strain, that harboured wMel [18], and four conplastic strains which had been produced by 20 backcrosses of Bi90^T^ males with appropriate source of *Wolbachia*. *Wolbachia* donor strains were also characterized for infection (wMelCS, wMel2, wMel4 and wMelPop) and mtDNA [18, 22, 41] (Table 1). Two independent runs were performed to make each conplastic strain, and finally two strains of ‘certain Wolbachia’-cytoplasmic/Bi90 nuclear background were created.

**Table 1:** Sources of ‘certain *Wolbachia’* infections used in the study

*Drosophila* strain	*Wolbachia* infection	Donor of cytoplasm	Origin of donor strain
Bi90^T^	non-infected	Bi90, tetracycline treated for 3 generations	Kyrgystan, 2004
Bi90^Mel^	wMel	Bi90	Kyrgystan, 2004
Bi90^Mel2^	wMel2	42	Yuzhno-Sakhalinsk, 2015
Bi90^Mel4^	wMel4	w304	Sinai Peninsula, Egypt, 2010
Bi90^CS^	wMelCS	w153	Uzbekistan, 1989
Bi90^Pop^	wMelPop	Iso wmelPop (high copy)	courtesy of Luis Teixeira (Instituto Gulbenkian de Ciência, Oeiras, Portugal)

All strains were kept at 25 °C, 12:12 h photoperiod, in a standard *Drosophila* medium (agar-agar, 7 g L^−1^; corn grits, 50 g L^−1^; dry yeast, 18 g L^−1^; sugar, 40 g L^−1^). Flies hatched within 3–4 h were pooled for experiments.

### Viability analysis

Viability analysis under heat stress was designed as follows: 1 day before the experiment, females were separated from males, and 5 flies were placed in a vial (25–48 vials in each group under study). Before and after the experiments, the flies were kept at 25 °C. To determine the viability under heat stress, the vials with flies were transferred from 25° to 38 °C for 4 h, and then were returned to 25 °C. 24 h later surviving flies were counted and survival rates were calculated as the percentage of survivors in each vial.

To estimate the effect of L-dihydroxyphenylalanine (L-DOPA) treatment on the stability against heat stress, five 4-day-old female flies were placed in vials (17–42 vials in each group under study) in which the bottom and 1 cm of the wall were covered with filter paper soaked with 0.5 mL of the nutrition medium. The medium contained 5% sucrose, 2% yeast and 1% L-DOPA (Sigma-Aldrich, USA). After 48 h, the vials were transferred from 25 °C to 38 °C for 2 h 45 min, and then returned to 25 °C. Survivors were counted in 24 h.

### Enzyme activity assays

To perform ALP and DAT activity measurements a spectrophotometric method was used. To measure ALP activity, flies (10–50 in each group under study) were homogenised on ice in 0.1 M Tris-phosphate buffer (Sigma-Aldrich, USA), pH 8.6 (1 fly in 20 μl) and centrifuged for 5 min at 13,030 g. Enzyme activity in the supernatant was determined using α-naphthylphosphate as substrate. After centrifugation, the supernatant was transferred to Eppendorf microtube (1.5 ml, Axygen Inc., USA) to which 1 ml of reaction mixture (100 ml 0.1 M Tris-phosphate buffer, pH 8.6, 100 mg α-naphthylphosphate, 100 mg fast blue RR salt (Chemapol, Czech Republic), 230 μl 10% MnCl, 230 μl 10% MgCl, 0.5 g polyvinylpyrrolidone (ICN, Russia), and 2 g NaCl) was added. Incubation was carried out at room temperature in the dark for 25 min, and the reaction was interrupted by the addition of 3 ml of ice-cold distilled water.

To measure DAT activity, flies (10–38 in each group under study) were homogenised on ice in 0.05 M Tris-HCl buffer (Sigma-Aldrich, USA), pH 7.2 (2 flies in 120 μl) and centrifuged 5 min at 13,030 g. Enzyme activity in the supernatant was determined using DA (Sigma-Aldrich, Switzerland) as substrate. The components of the reaction mixture were added to a cuvette as follows: 300 μl of 0.05 M Tris-HCl, pH 7.2, 50 μl of acetyl CoA (0.5 mM, Sigma-Aldrich, USA) in 0.05 M Tris-HCl, pH 7.2, 25 μl of 12 mM N-phenylthiourea (Fluka, China) in 0.05 M Tris-HCl, pH 7.2, 25 μl of 40 mM DA in 0.001 N HCl, 50 μl of the supernatant, and 50 μl of 2.4 mM 5,5-dithiobis(2-nitrobenzoic acid) (Fluka, USA) in 0.05 M Tris, pH 7.2. The samples were incubated for 2 min at room temperature in the dark.

The optical density of the obtained reaction products was measured with a SmartSpec™ Plus spectrophotometer (Bio-Rad, USA) at 405 nm (DAT) and 470 nm (ALP) against the reaction zero point. For ALP activity measurements under heat stress flies were exposed to 38 °C for 1 h 40 min; the optimum exposure time was determined previously [36].

### Statistics

All data are represented as means ± S.E.M. The false-discovery rate corrections for multiple comparisons were made when appropriate. The data on ALP activity, DAT activity and fly viability were analyzed by 1-way ANOVA (Strain – the simple factor) or by 2-way ANOVA (Strain – the 1st simple factor; Heat stress or L-DOPA treatment – the 2nd simple factor). Before performing the ANOVA, a Shapiro-Wilk’s W test was used to assess normality of the datasets analyzed. All datasets that failed to meet the assumptions of the ANOVA were transformed prior to analysis. The comparison of the group means was performed with the Benjamini-Hochberg stepwise post-hoc test. The results were considered significant at probability level < 0.05.

## Results

### The heat stress impact on viability of *D. melanogaster* infected with different Wolbachia genotypes

**Fig. 1:**
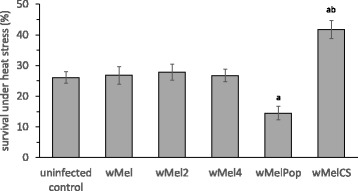
The effect of various *Wolbachia* infections on *Drosophila* heat stress resistance in comparison with uninfected (tetracycline-treated) control. The data represents survival rate of 6-day-old *Drosophila* females under 4 h of heat exposure (38^о^C). Each histogram bar represents an average value of 25–48 tests (means ± SEM). a – *p* < 0.01 vs uninfected and *wMel, wMel2, wMel4* infected groups. b – *p* < 0.0001 vs *wMelPop* infected group

The results of an evaluation of the viability after heat stress exposure (4 h 38 °C) of 6-day-old *Drosophila* females of wild type strain Bi90^T^ (uninfected control) and strains that harboured *wMel, wMel2, wMel4, wMelCS* and *wMelPop Wolbachia* variants are presented in Fig. 1. No significant difference was found in the survival rates under heat stress between females with *Wolbachia* genotypes *wMel*, *wMel2* and *wMel4* and uninfected control. On the contrary, the survival of females with *wMelPop* infection was significantly decreased compared with control females and females with *wMel, wMel2* and *wMel4* genotypes, whereas females with *wMelCS* genotype of *Wolbachia* demonstrated a significant increase of viability under heat stress (Fig. 1; Strain – F_(5211)_ = 14.05, *p* ≪ 0.00001).

### The effects of various Wolbachia genotypes on *D. melanogaster* alkaline phosphatase (ALP) activity

**Fig. 2:**
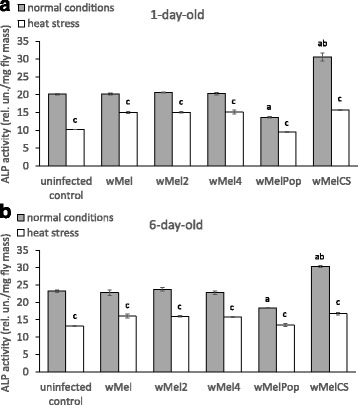
The effect of various *Wolbachia* infections on ALP activity in comparison with uninfected (tetracycline-treated) control. **a** 1-day-old and **b** 6-day-old *Drosophila* females under normal conditions and upon heat stress (38^о^C). Each histogram bar represents an average of 10 to 50 measurements (means ± SEM). a – *p* < 0.001 vs uninfected and *wMel, wMel2, wMel4* infected groups that were not stressed. b – *p* < 0.0001 vs *wMelPop* infected non-stressed group. c – *p* < 0.001 vs the control non-stressed group of the same strain

ALP converts the inert tyrosine conjugate, tyrosine-O-phosphate, into tyrosine and thus changes in ALP activity usually correlate with changes in the DA level in flies [35, 38]. The ALP activity of 1- and 6-day-old *Drosophila* females infected with *wMel, wMel2, wMel4, wMelCS* and *wMelPop Wolbachia* variants and uninfected Bi90^T^ strain were measured under normal and heat stress (1 h 40 min 38 °C) conditions. No statistical significance under normal conditions between the ALP activities of the control uninfected flies and flies with *Wolbachia* genotypes *wMel*, *wMel2* and *wMel4* was found (Fig. 2a, b). However, the ALP activities in 1- and 6-day-old flies with *wMelPop* infection were lower and in 1- and 6-day-old flies with *wMelCS* – higher, than in *wMel*, *wMel2, wMel4* and Bi90^T^ at the same age (for Day 1 – Fig. 2a; Strain – F_(5213)_ = 269.41, *p* ≪ 0.00001; for Day 6 – Fig. 2b; Strain – F_(5233)_ = 56.87, *p* ≪ 0.00001). The significant decrease in ALP activity following heat stress in the females of both ages of every strain under study was demonstrated (for Day 1 – Fig. 2a; Stress – F_(1213)_ = 1270.47, *p* ≪ 0.00001; Strain*Stress – F_(5213)_ = 74.22, *p* ≪ 0.00001; for Day 6 – Fig. 2b; Stress – F_(1233)_ = 867.15, *p* ≪ 0.00001; Strain*Stress – F_(5233)_ = 22.22, *p* ≪ 0.00001).

### The effects of different Wolbachia genotypes on *D. melanogaster* dopamine-dependent arylalkylamine N-acetyltransferase (DAT) activity

**Fig. 3:**
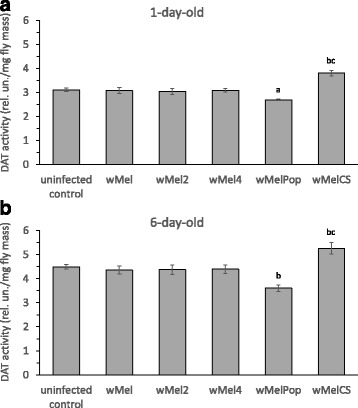
The effect of various *Wolbachia* infections on DAT activity in comparison with uninfected (tetracycline-treated) control. **a** 1-day-old and **b** 6-day-old *Drosophila* females. Each histogram bar represents an average of 10 to 38 measurements (means ± SEM). a – *p* < 0.05 vs uninfected and *wMel, wMel2, wMel4* infected groups. b – *p* < 0.01 vs uninfected and *wMel, wMel2, wMel4* infected groups. c – *p* < 0.0001 vs *wMelPop* infected group

DAT also takes a part in the regulation of DA content in flies [37], so we measured DAT activities in 1- and 6-day-old *Drosophila* females infected with *wMel, wMel2, wMel4, wMelCS* and *wMelPop Wolbachia* variants, as well as in the uninfected Bi90^T^ females (Fig. 3). Since DAT does not respond to stress in *Drosophila* [42], we measured its activity under normal conditions only. The *wMelPop* infection results in a significant decrease and *wMelCS* – in an increase of DAT activity compared with the control in both ages (for Day 1 – Fig. 3a; Strain – F_(5147)_ = 16.06, *p* ≪ 0.00001; for Day 6 – Fig. 3b; Strain – F_(5107)_ = 16.61, *p* ≪ 0.00001). Other *Wolbachia* variants under study do not affect DAT activity in either 1- or 6-day-old females. DAT determines, at least particularly, the basal level of DA [37, 38], so we have assumed that *Wolbachia* infection affects it.

### The influence of dopamine level on the survival under heat stress of females infected with different Wolbachia variants

**Fig. 4:**
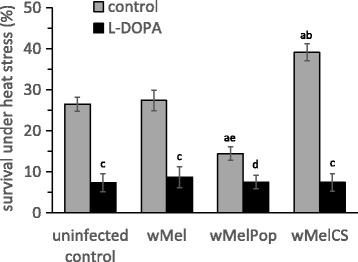
The effect of L-DOPA on heat stress resistance of *Drosophila* females with various *Wolbachia* infections in comparison with uninfected (tetracycline-treated) control. The data represents survival rate of 6-day-old *Drosophila* females under 4 h of heat exposure (38^о^C) following 2 days of L-DOPA treatment. L-DOPA designates the uninfected flies and flies with various *Wolbachia* infections that were treated with L-dihydroxyphenylalanine. Each histogram bar represents an average value of 17–42 tests (means ± SEM). a – *p* < 0.001 vs uninfected and *wMel* infected groups that not received L-DOPA. b – *p* < 0.0001 vs *wMelPop* infected group that not received L-DOPA. c – *p* < 0.0001 vs the control group of the same strain that not received L-DOPA. d – *p* < 0.05 vs the control group of the same strain that not received L-DOPA. e – *p* < 0.05 vs uninfected and *wMel, wMelCS* infected groups that received L-DOPA

To find out whether the changes in the heat stress resistance of *Drosophila* females infected with *wMelPop* and *wMelCS* have a connection with the altered DA level, we examined their stress resistance following a pharmacological increase of DA content. Feeding with L-DOPA was shown to double the DA level in *Drosophila* [36], so we fed these females as well as infected with *wMel* and uninfected control with L-DOPA for 48 h before stress exposure. The rise of the DA level decreases the survival of all four groups under heat stress and eliminates the differences between the survival rate of control and *wMel* females and that of females infected with *wMelPop* and *wMelCS* (Fig. 4; Strain – F_(3222)_ = 14.79, *p* ≪ 0.00001; L-DOPA – F_(1222)_ = 174.18, *p* ≪ 0.00001; Strain*L-DOPA – F_(3222)_ = 14.86, *p* ≪ 0.00001). It is worth noting that the survival rates in Bi90^T^, *wMel* and *wMelCS* females after L-DOPA feeding do not differ in this parameter from the L-DOPA-treated females with *wMelPop* infection (Fig. 4). The survival rates in *wMelPop* L-DOPA untreated females were low but still higher than in Bi90^T^, *wMel* and *wMelCS* females after L-DOPA feeding (Fig. 4).

## Discussion

Here we try to reveal the influence of the *Wolbachia* symbiont on heat stress resistance and DA metabolism in *D. melanogaster*. This investigation was motivated by the reports on *Wolbachia* effect on insulin signaling [11, 43] and data on *Wolbachia* diversity in *D. melanogaster* [18, 20–22, 27]. Ikeya et al. [11] had demonstrated the increase of insulin signaling in *Wolbachia*-infected strains. Insulin signaling pathway interacts with the components of the neuroendocrine stress reaction and the stress-responsive c-Jun-N-terminal kinase (JNK) signaling pathway (which controls of a large number of cellular processes in response a wide range of stressors), and contribute to the fitness and increased stress tolerance [29, 30, 44–46]. The removal of *Wolbachia* from *chico* ^*2*^ homozygotes (*chico* gene codes the *Drosophila* orthologue of mammalian insulin receptor substrate) resulted in complete lethality [43]. *Wolbachia* infection was also shown to down-regulate 41% (11 of 27) of known heat shock proteins in the *Drosophila* S2 cell line [47].

Using data on *D. melanogaster* strains with uniform nuclear background but infected with different *Wolbachia* variants we have shown that *Wolbachia* genotypes *wMel*, *wMel2* and *wMel4* (of V, VIII, and III clade, respectively) do not induce alteration in the heat stress resistance and DA metabolism of the host.

However, two *Wolbachia* isolates under study do cause the changes in survival rate and DA metabolism of *D. melanogaster* host: *wMelPop* and *wMelCS*. The *wMelPop* infection reduces both the survival and activities of ALP and DAT, whereas the *wMelCS* infection increases these parameters (see Figs. 2 and 3). Previously, we found the negative correlation of the heat stress resistance with the DA level in *Drosophila* [48]. The DA level in *D. melanogaster* is negatively correlated with the level of ALP and DAT activities [36, 38]. Based on this observation, we assumed the DA level to be decreased in the flies with *wMelCS* infection (and to be increased in the flies with *wMelPop*). We verified this assumption using the treatment of the flies with the DA precursor, L-DOPA (see Fig. 4). The increase of DA level drastically reduces the survival rates of all studied strains. It is important that increased DA has been revealed to level the viability under heat stress of *wMelCS*-infected flies with other strains (see Fig. 4).

Low survival under heat stress of the *wMelPop*-infected flies could be explained by the well-known pathogenicity of this *Wolbachia* strain [23, 49]. But it is noteworthy that the changes in the DA metabolism are manifested in these flies prior to the mass death of flies (see Figs. 2a and 3a) [23]. The negative effect of *wMelPop* (in comparison with *wMel-* and *wMelCS-*infected and uninfected strains) on the level and the biosynthesis of one more biogenic amine involved in the stress reaction, octopamine, in *D. melanogaster* was shown by Rohrscheib et al. [50]. However, no difference in octopamine biosynthesis pathway was found between flies with *wMel* and *wMelCS Wolbachia* genotypes [50]. Perhaps this is due to various roles of DA and octopamine in flies, or with some delicate genetic differences in *Wolbachia* strains used in our study and in the study of Rohrscheib et al. [50].

We believe that the most interesting result, which we observed here, is the effect of *wMelCS Wolbachia* on *D. melanogaster* viability under stress and DA metabolism. Based on the study of Riegler et al. [22] who proposed the hypothesis of global replacement of *Wolbachia wMelCS* infection by *wMel* in *D. melanogaster* we expected to find a decreased fitness in *wMelCS*-infected flies compared with *wMel*-infected. However, we have found quite the opposite phenomenon.

The design of our study included an attempt to find phylogenetical signal of symbiont effects on the host. Previous works using genome data for both mtDNA and *Wolbachia* have revealed strict associations of those maternal factors and have distinguished coevolved clades of *Wolbachia* and mtDNA in *D. melanogaster* [18, 20, 21, 27]. Here we showed the influence of *wMelCS,* but not *wMel*-like, *Wolbachia* variants on the components of host fitness. The *wMelCS*-like isolates are related to *Wolbachia* clade VI that is the most diverged from all other clades, the time divergence of *wMel* and *wMelCS*-like variants is approximately in range 3.2–14 Kya [20]. Thus, we assume a specific influence of *Wolbachia* clade VI on *D. melanogaster* that should be verified in the following experiments.

## Conclusions

Here we revealed that the effect of *Wolbachia* symbiont on the stress resistance and DA metabolism of the host insect depends on the symbiont’s genotype variant. We found out that *wMelCS* genotype demonstrates a strong positive influence on the *D. melanogaster* heat stress resistance, while survival rates of the flies with *Wolbachia* genotypes of *wMel* group do not differ from those of uninfected flies. This result is particularly surprising because genotypes of *wMel* group predominate in the nature populations all over the world and *wMelCS* variants are very rare. It is necessary to check whether such a fitness effect is inherent in all *wMelCS* variants or we are faced with a particular case similar to that of pathogenic wMelPop strain only “with an opposite sign”. Besides, we discovered that strong influence of wMelPop strain on *D. melanogaster* metabolism starts much earlier than mass death of flies.

## Abbreviations

ALP: Alkaline phosphatase
DA: Dopamine
DAT: DA-dependent arylalkylamine N-acetyltransferase
L-DOPA: L-dihydroxyphenylalanine
